# Genomic Epidemiology in Filarial Nematodes: Transforming the Basis for Elimination Program Decisions

**DOI:** 10.3389/fgene.2019.01282

**Published:** 2020-01-09

**Authors:** Shannon M. Hedtke, Annette C. Kuesel, Katie E. Crawford, Patricia M. Graves, Michel Boussinesq, Colleen L. Lau, Daniel A. Boakye, Warwick N. Grant

**Affiliations:** ^1^Department of Physiology, Anatomy and Microbiology, La Trobe University, Bundoora, VIC, Australia; ^2^Unicef/UNDP/World Bank/World Health Organization Special Programme for Research and Training in Tropical Diseases (TDR), World Health Organization, Geneva, Switzerland; ^3^College of Public Health, Medical and Veterinary Sciences, James Cook University, Cairns, QLD, Australia; ^4^Unité Mixte Internationale 233 "TransVIHMI", Institut de Recherche pour le Développement (IRD), INSERM U1175, University of Montpellier, Montpellier, France; ^5^Department of Global Health, Research School of Population Health, Australian National University, Acton, ACT, Australia; ^6^Parasitology Department, Noguchi Memorial Institute for Medical Research, Accra, Ghana

**Keywords:** population genomics, onchocerciasis, lymphatic filariasis, transmission, parasite elimination, drug resistance, epidemiology

## Abstract

Onchocerciasis and lymphatic filariasis are targeted for elimination, primarily using mass drug administration at the country and community levels. Elimination of transmission is the onchocerciasis target and global elimination as a public health problem is the end point for lymphatic filariasis. Where program duration, treatment coverage, and compliance are sufficiently high, elimination is achievable for both parasites within defined geographic areas. However, transmission has re-emerged after apparent elimination in some areas, and in others has continued despite years of mass drug treatment. A critical question is whether this re-emergence and/or persistence of transmission is due to persistence of local parasites—i.e., the result of insufficient duration or drug coverage, poor parasite response to the drugs, or inadequate methods of assessment and/or criteria for determining when to stop treatment—or due to re-introduction of parasites *via* human or vector movement from another endemic area. We review recent genetics-based research exploring these questions in *Onchocerca volvulus*, the filarial nematode that causes onchocerciasis, and *Wuchereria bancrofti*, the major pathogen for lymphatic filariasis. We focus in particular on the combination of genomic epidemiology and genome-wide associations to delineate transmission zones and distinguish between local and introduced parasites as the source of resurgence or continuing transmission, and to identify genetic markers associated with parasite response to chemotherapy. Our ultimate goal is to assist elimination efforts by developing easy-to-use tools that incorporate genetic information about transmission and drug response for more effective mass drug distribution, surveillance strategies, and decisions on when to stop interventions to improve sustainability of elimination.

## Introduction

Onchocerciasis and lymphatic filariasis (LF) are targeted for elimination, primarily using community-wide mass drug administration (MDA). In April 2019, the World Health Organization (WHO) initiated a global consultation on the “Roadmap for Neglected Tropical Diseases,” including current targets for global elimination of LF as a public health problem by 2020 (World Health Organozation, 2012a) and elimination of transmission of the parasite that causes onchocerciasis in 80% of affected sub-Saharan countries by 2025 (African Programme for Onchocerciasis Control, 2012). Barriers to elimination include operational challenges for adequate drug distribution or non-compliance leading to low treatment coverage (Churcher et al., 2009; Turner et al., 2013;  African Programme for Onchocerciasis Control, 2015), movement of humans or vectors among communities (De Sole et al., 1991; African Programme for Onchocerciasis Control and World Health Organization, 2010; Ramaiah, 2013; Xu et al., 2018b; *Delineating Transmission Zones for Sustainable Onchocerciasis and LF Elimination*), and challenges in determining when the transmission cycle has been interrupted and treatment can be stopped (Stolk et al., 2015; Lont et al., 2017; Walker et al., 2017). Elimination of these diseases has been formally verified or validated by the WHO in some geographic regions, and is under assessment in others (*Parasite Transmission, Morbidity, and Control/Elimination Strategies*). However, there are areas where transmission has re-emerged after apparent (though not WHO-certified) elimination, and others where transmission has continued despite decades of MDA. A critical question is whether “local” parasites continue to persist because of insufficient MDA coverage and/or duration relative to the pre-intervention endemicity, poor parasite drug response, or inadequate methods of assessment and/or criteria for decisions to stop treatment, or whether “immigrant” parasites have been introduced *via* human or vector movement from another endemic area. Population genetic analyses of parasite DNA sequences may provide data to answer this question. With the increasing technical feasibility of gathering population-level genomic data, it becomes possible to develop tools allowing routine acquisition of such data by elimination programs to inform decisions on MDA strategies, delineate areas to be included in evaluations, and determine whether treatment should be continued or stopped.

### Parasite Transmission, Morbidity, and Control/Elimination Strategies

#### Onchocerciasis

**Figure 1:**
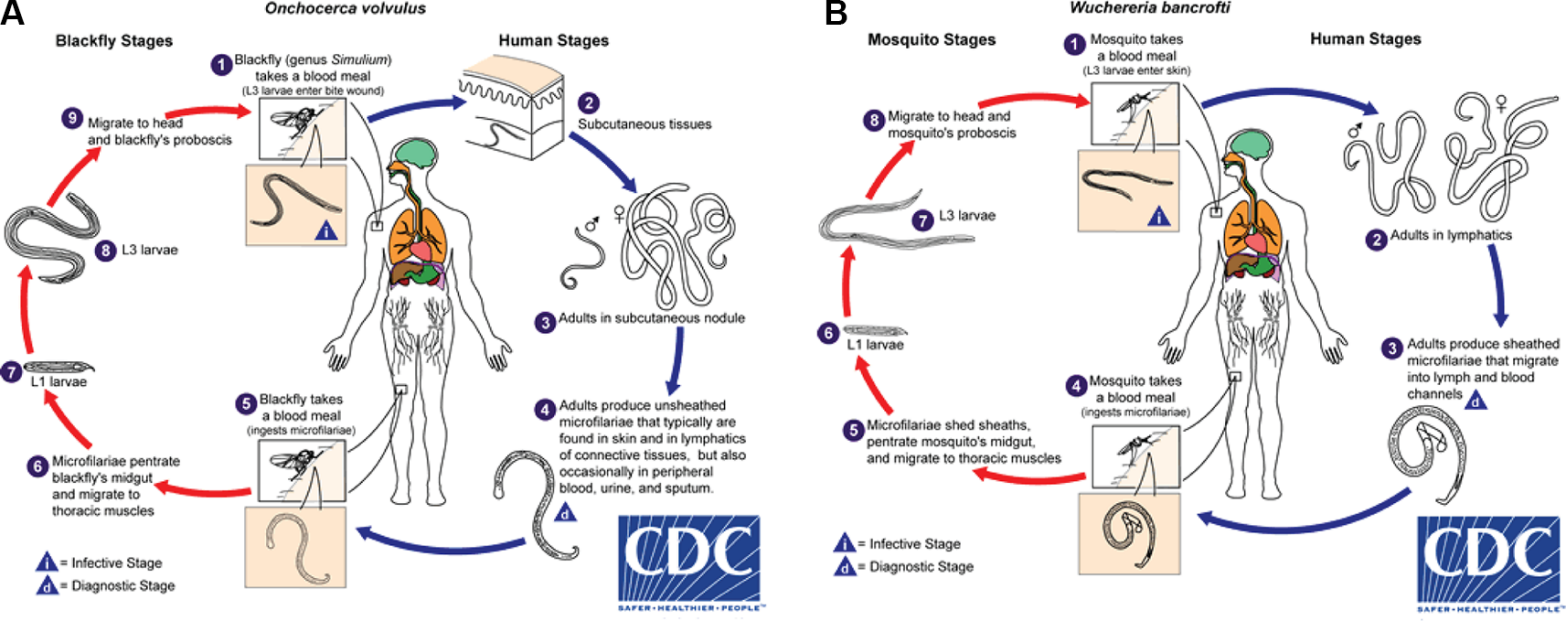
Life cycles of **(A)** *Onchocerca volvulus* and **(B)** *Wuchereria bancrofti*. From Centers for Disease Control and Prevention (accessed April 2019, https://www.cdc.gov/dpdx/az.html).

*Onchocerca volvulus* is transmitted by blackflies of the genus *Simulium*. Female blackflies taking a blood meal from an infected person ingest *O. volvulus* microfilariae, which develop into infective L3 larvae that are transmitted to a different host during subsequent blood meals (**Figure 1A**). The L3 larvae develop *via* L4 into adult worms, macrofilariae, which live for around 12–14 years in nodules under the skin and deep in the body, producing millions of microfilariae. The microfilariae migrate through the skin, eyes, and other organs, and live for up to 2 years. The immunological reactions upon death of microfilariae are the major cause of morbidity: primarily itching, skin depigmentation and lesions, and visual impairment that can progress to blindness (Remme et al., 2017). Increasing evidence suggests that high *O. volvulus* infection can induce epilepsy (Chesnais et al., 2018).

The pattern of onchocerciasis endemicity is determined by ecological conditions favorable for *Simulium* breeding sites, whose location and productivity (i.e., number of blackflies hatching) can vary seasonally and from year to year. *Simulium damnosum sensu lato* (*s.l.*), the main vector in Africa, breeds in fast-flowing rivers and streams with the high level of oxygenation needed for larval development. The flies typically have an active flight range of around 15 km, but can migrate seasonally over hundreds of kilometers if assisted by prevailing winds, and thus transmit the parasite over large geographic ranges (Garms et al., 1979; Le Berre et al., 1979; Baker et al., 1990). Infection rates are highest among those living and/or working close to consistently highly productive breeding sites and decline with increasing distance between where blackflies breed and where people live and work.

The morbidity and socio-economic impact of onchocerciasis motivated three control programs. The Onchocerciasis Control Programme in West Africa (OCP, 1974–2002) targeted elimination as a public health problem through weekly aerial larviciding of breeding sites along ~50,000 km of rivers for the life span of macrofilariae, later complemented in some areas by mass drug administration of ivermectin (MDAi). The OCP originally covered ~654,000 km^2^, which was expanded to 1,300,000 km^2^ because *Simulium* from outside invaded the original area (Le Berre et al., 1979; Baker et al., 1990; Remme, 2004; Boatin, 2008).

**Figure 2:**
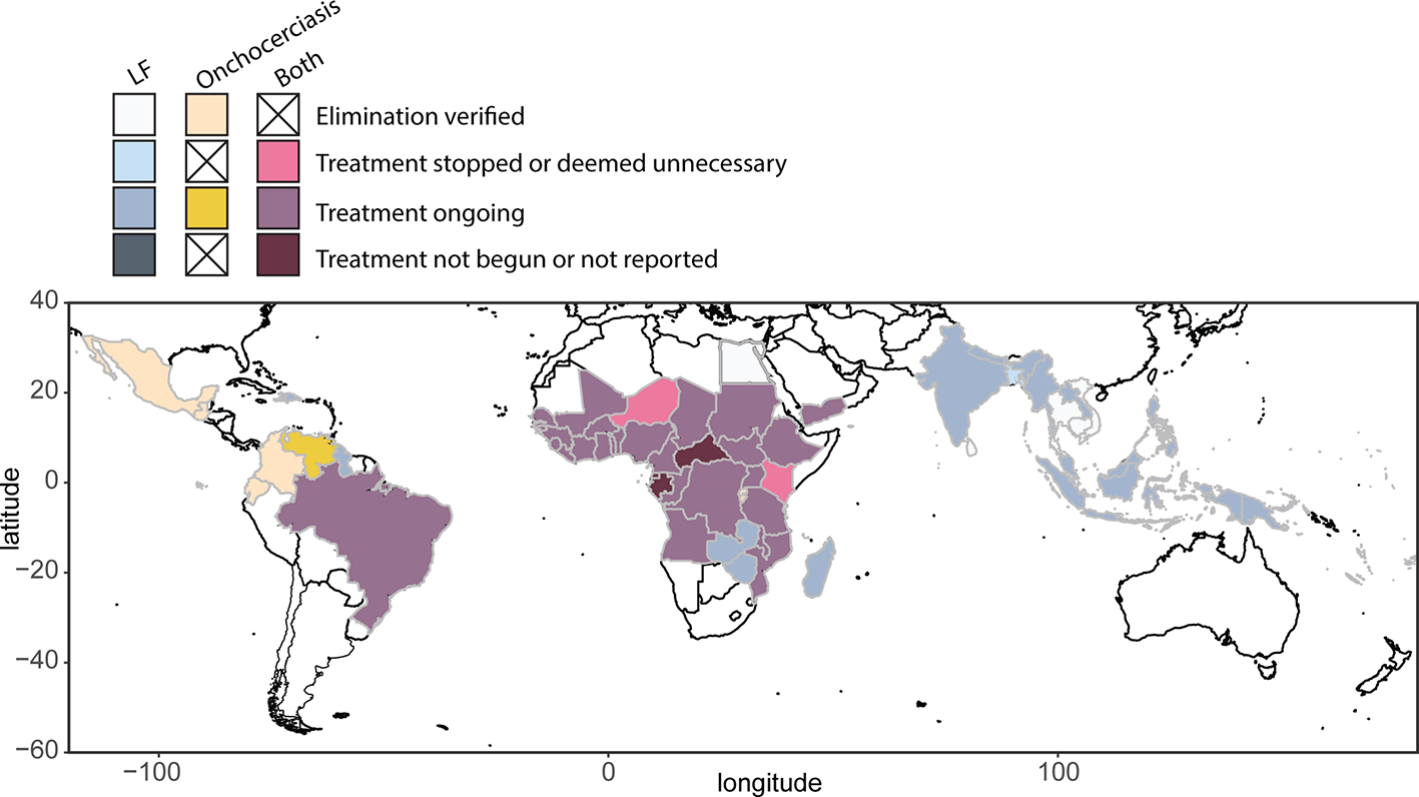
Global distribution and treatment of lymphatic filariasis (2018) and onchocerciasis (2017), based on WHO Global Health Observatory data repository (accessed April 2019, https://www.who.int/gho/en/).

The 1987 decision of Merck & Co., Inc. (Kenilworth, NJ, USA) to donate ivermectin (Mectizan^®^) for onchocerciasis control, and subsequent studies showing the safety of MDAi, allowed initiation of MDAi-based control programs. The Onchocerciasis Elimination Program for the Americas (OEPA) was initiated in 1993 to eliminate transmission in 13 generally small foci across six countries with a total at-risk population of 0.56 million. Building on early efforts to control onchocerciasis through nodulectomy campaigns and annual ivermectin treatments, the OEPA implemented health system–directed biannual MDAi (and even quarterly MDAi in four areas in Brazil, Mexico, and Venezuela). Elimination was certified by WHO for all but the large Amazonian area across the border between Brazil and Venezuela, where difficult terrain and lack of roads make it challenging to ensure ivermectin distribution to ~30,500 Yanomami across ~540 migratory communities (Sauerbrey et al., 2018; World Health Organization, 2018; **Figure 2**).

**Figure 3:**
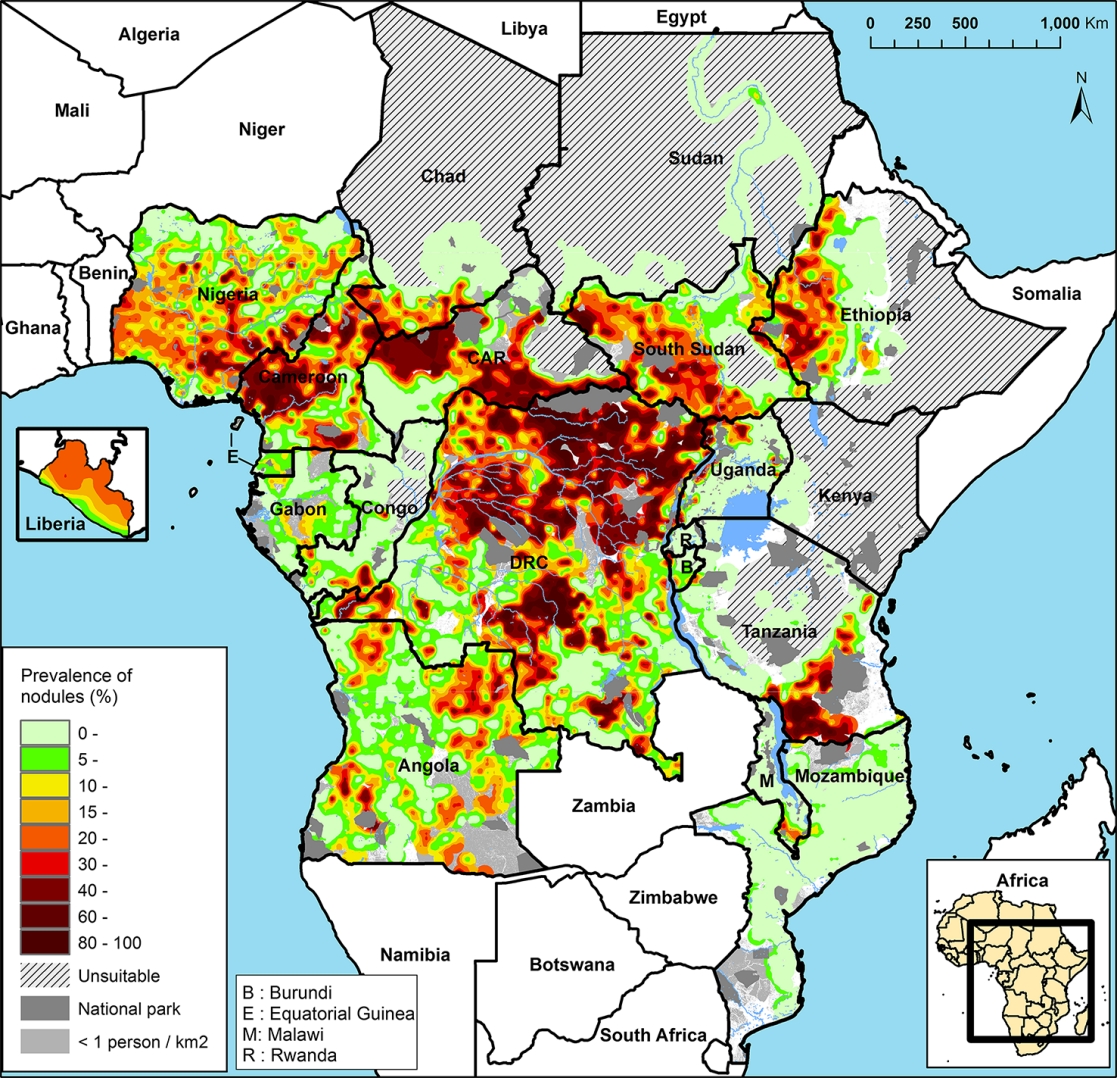
Rapid epidemiological mapping of onchocerciasis (REMO), based on prevalence of nodules across communities with a history of onchocerciasis and covered by the African Programme for Onchocerciasis Control (APOC). Figure from Zouré et al., (2014).

The APOC (1995–2015) initially targeted control of onchocerciasis as a public health problem in Central and East Africa and Liberia with sustainable, community-directed ivermectin treatment (CDTI). Across the 19 APOC countries, ~86 million people were estimated to require CDTI because they lived in meso- and hyperendemic areas, i.e., where 20–40% and >40% of adults, respectively, have subcutaneous nodules (Noma et al., 2014; **Figure 3**). These nodule prevalences correspond to prevalences of skin microfilariae in the general population of around 40–60% for mesoendemic and >60% for hyperendemic areas associated with an increased risk of onchocercal blindness (Prost et al., 1979; UNDP/World Bank/WHO Special Programme for Research and Training in Tropical Diseases, 1992; Noma et al., 2002; Seketeli, 2002; Seketeli et al., 2002; Fobi et al., 2015). The sizes of these areas range from relatively small to a vast contiguous endemic area of ~2 million km^2^ across seven countries (Noma et al., 2014; World Health Organization, 2018). Research and subsequent epidemiological evaluations of infection rates in areas with long-term MDAi (Diawara et al., 2009; Traore et al., 2012; Tekle et al., 2016) suggested that MDAi alone could achieve elimination of transmission in Africa. The APOC objectives were consequently expanded to onchocerciasis elimination in at least 80% of African countries by 2025 (African Programme Onchocerciasis Control, 2012). In 2015, APOC was closed. The Expanded Special Project for Elimination of Neglected Tropical Diseases (ESPEN) now provides countries with some of the support previously provided by APOC.

MDAi in meso- or hyperendemic areas may have reduced infection prevalence in neighboring hypoendemic areas. Previously unmapped hypoendemic areas that might impact elimination goals (Rebollo et al., 2018) by acting as a potential source of renewed transmission need to be efficiently determined and cost-effective intervention strategies implemented in these areas (and those previously identified as hypoendemic and not included in CDTI). Including the population in hypoendemic areas mapped by 2017–2018, WHO estimated that 204 million people required MDAi to achieve elimination (World Health Organization, 2018). In Central Africa where another filarial parasite, *Loa loa*, is endemic, areas previously identified as requiring interventions based on parasites observed in the skin may require remapping. Recent data suggest that *L. loa* microfilariae, once assumed to be present only in blood, may also be present in the skin and mistaken for *O. volvulus* (Nana-Djeunga et al., 2019).

The OCP experience (Remme et al., 1995) informed the 2001 WHO procedures for entomological assessments and criteria for certification of onchocerciasis elimination (World Health Organization, 2001). Those procedures stipulated testing at least 10,000 flies from each endemic community selected for evaluation by the WHO International Certification Team, maximizing the chances of detecting infected flies by collecting during peak transmission season. In 2010, APOC outlined a “Conceptual Framework of Onchocerciasis Elimination with Ivermectin Treatment” which built on the OCP and APOC experience, took into account differences between control *via* vector elimination and *via* ivermectin treatment, and emphasized that much is still to be learned (African Programme for Onchocerciasis Control/World Health Organization, 2010; Dadzie et al., 2018). This framework defined elimination as “reduction of infection and transmission to the extent that interventions can be stopped but post-intervention surveillance is still necessary” and a transmission zone as a “geographical area where transmission of *O. volvulus* occurs by locally breeding vectors and which can be regarded as a natural ecological and epidemiological unit for interventions.” It identified delineating transmission zones and examining risks of parasite reintroduction through human or vector migration as critical to decisions to stop MDA. Acknowledging that “in practice it is difficult to determine with a fair degree of certainty that vectors in a given area are exclusively locally breeding,” the framework recommended an operational definition of a transmission zone as a “river basin, or a major section of a river basin, where onchocerciasis is endemic and where the river is the core of the endemic area, with communities with the highest prevalence of infection generally located close to the river and infection levels falling with increasing distance from the river until they become negligible or reach a neighboring transmission zone.” In 2016, the WHO issued a new guideline for stopping MDA and verifying elimination of human onchocerciasis (World Health Organization, 2016). This guideline defines interruption of transmission of *O. volvulus* as “the permanent reduction of transmission in a defined geographical area after all the adult worms (and microfilariae) in the human population in that area have died, been exterminated by some other intervention, or become sterile and infertile.” While this guideline does not specify criteria for a “defined geographical area,” it defines a transmission zone in the same way as the APOC framework. The 2016 Guideline Development Group recommended that decisions about interruption of parasite transmission, stopping MDA, and confirmation of transmission interruption at the end of the post-treatment surveillance period should be based on a prevalence of infective parous flies of <0.1%. Statistical confidence for this elimination threshold requires that a minimum of 6,000 flies collected from “a transmission zone” should be tested. The guideline does not address delineation of a transmission zone and hence the geographical range over which those flies are to be collected. To date, no validated methodology for transmission zone delineation is available. We review evidence here that population genetic measures of the parasite (and its vectors) capture the history of transmission and hence can be used as a proxy for defining transmission zone boundaries.

#### Lymphatic Filariasis

LF is a vector-borne disease caused by three species of parasites: *Wuchereria bancrofti, Brugia malayi*, and *Brugia timori*. The majority of infections are due to *W. bancrofti*, which is found throughout the tropics (**Figure 2**). Chronic infection with these parasites can disrupt lymphatic function and lead to long-term disability due to lymphedema (most commonly swollen lower limbs) and scrotal hydrocele (Taylor et al., 2010; World Health Organzation, 2011). Globally, ~68 million are infected with LF, with 36 million microfilaremic persons, 19 million hydrocele cases, and 17 million lymphedema cases (Ramaiah and Ottesen, 2014). In 1997, following a World Health Assembly resolution, the WHO initiated elimination of LF as a public health problem, using a two-pillar program of annual MDA to reduce microfilaremia to levels that would not sustain continued transmission, and management of morbidity and disability by providing a minimum package of care (Ichimori et al., 2014).

Infective larval LF are transmitted to humans by mosquitoes and migrate to the lymphatic system where they develop into adult worms that live for ~4 to 6 years (**Figure 1B**). After mating, female adult worms produce thousands of microfilariae throughout their life, which circulate in the peripheral blood and are available to infect mosquitoes during blood meals to continue the transmission cycle. LF is transmitted by a wide range of mosquito genera including *Anopheles*, *Culex*, *Mansonia*, and *Aedes* (World Health Organization, 2013). In a striking example of the power of adaptive evolutionary change, the daily periodicity in density of microfilariae in peripheral blood is tuned to the biting times of local vectors.

Human infections are diagnosed by detecting microfilariae on blood films collected at the appropriate time of day, or by detecting circulating filarial antibodies or antigens (Ag). Detecting microfilariae on blood films requires technical skill and equipment and collecting blood at night (where vectors are nocturnal) is challenging, so serology is usually used rather than microscopy. The current standard test for programmatic surveillance activities in *W. bancrofti* areas is a rapid diagnostic test for circulating filarial antigens (World Health Organization, 2017b). Antibody tests are not usually used because antibodies can persist for years after the death of adult worms. Both overall and age-specific prevalence of LF infection (usually not peaking until adulthood) is determined by transmission intensity, which in turn depends on vector biting rates and the availability of susceptible humans. The threshold for starting MDA programs is microfilariae or antigen prevalence of ≥1% within an implementation unit (IU), which is defined prior to mapping (World Health Organization, 2011; World Health Organization, 2017b). The size of the IU was left to the judgment of the endemic countries; some chose to map by village or district, while others classified the entire country or regions. In areas in Africa co-endemic for loiasis (*L. loa* filariasis), the use of antigen-based test strips for mapping has probably overestimated LF prevalence, because of false positives from people with high intensity infection with *L. loa* (Wanji et al., 2015; Pion et al., 2016). Thus, mapping should be revisited in loiasis co-endemic areas using approaches that increase species specificity.

MDA is conducted annually with a single-dose combination of albendazole with diethylcarbamazine (DEC) or, in onchocerciasis co-endemic countries, with ivermectin. Recently, the three drugs have been combined in some countries (King et al., 2018), including those where elimination targets have not been reached despite completing the required rounds of two-drug MDA (World Health Organization, 2017a). In loiasis co-endemic areas in Central Africa not meso- or hyperendemic for onchocerciasis, biannual albendazole treatment in combination with vector management is recommended, since DEC and ivermectin can cause serious adverse reactions in individuals with high *L. loa* microfilaremia (Boussinesq et al., 2003; World Health Organization, 2012b).

The WHO recommendation is for MDA to be conducted until large, well-designed transmission assessment surveys (TASs) of 6- to 7-year-old children in schools or communities determine that the estimated antigen prevalence in an evaluation unit has dropped below a critical cutoff value, when transmission is not considered sustainable (World Health Organization, 2011; World Health Organization, 2017b). Critical cutoff values depend on the filarial and vector species, and are calculated such that the likelihood of an evaluation unit passing is at least 75% if true antigen prevalence is 0.5%, and no more than 5% if the true antigen prevalence is 1% (World Health Organization, 2011; World Health Organization, 2017b). For example, in regions where *W. bancrofti* is endemic and transmission is dominated by *Aedes* spp. mosquitoes, the threshold is based on an upper 95% confidence limit for antigen prevalence of 1%. The TAS evaluation unit can be the same as the IU, it can be larger and include several IUs, or it can be smaller (by splitting an IU), depending on available information on likely residual LF endemicity. Once a country has passed two post-MDA TASs at 2- to 3-year intervals in all evaluation units, it can apply for validation of elimination of LF as a public health problem. Post-validation surveillance also uses TAS in 6- to 7-year olds (World Health Organization, 2017b). Morbidity management and rehabilitation must continue as needed.

### Delineating Transmission Zones for Sustainable Onchocerciasis and LF Elimination

#### Onchocerciasis

The mosaic of onchocerciasis hyper-, meso-, and hypoendemicity in Africa implies an underlying geographic mosaic of parasite transmission zones (**Figure 3**; African Programme for Onchocerciasis Control/World Health Organization, 2010) that is the product of long-term, historical spatial density and migration patterns of the blackfly vector and of the human host (Blouin et al., 1995; Nadler, 1995; Jarne and Théron, 2001; Criscione and Blouin, 2004; Criscione et al., 2005; Prugnolle et al., 2005; Barrett et al., 2008). This pattern was not important for implementation of “project areas” for control of onchocerciasis as a public health problem (i.e., morbidity reduction and prevention), which were delineated based on the administrative borders of health system units in which meso- and hyperendemic areas were located. However, with the switch from control as a public health burden to elimination of transmission, explicit consideration should be given to the alignment of intervention areas and the areas included in the evaluations for decisions to stop treatment (subsequently referred to as “evaluation areas”) with the true, long-term parasite transmission zones.

Although the success of MDAi has driven the move from control to elimination of onchocerciasis, there are some areas in Africa in which many years of MDAi have reduced infection prevalence less than expected (i.e., less than transmission models predict) or where resumption of transmission has been reported following cessation of interventions (Kamga et al., 2016; Tekle et al., 2016; Koala et al., 2017; Bakajika et al., 2018; Komlan et al., 2018; Koala et al., 2019). Transmission models predict that the duration of interventions required to interrupt transmission is dependent on vector abundance (which determines the percentage of the population infected before interventions begin), and the treatment frequency and percentage of the population taking ivermectin (African Programme for Onchocerciasis Control/World Health Organization, 2010; Stolk et al., 2015). Consequently, the apparent failure of MDAi to interrupt transmission in some treatment areas, and of post-treatment recrudescence in others, might be due to highly hyperendemic areas requiring longer-than-assumed duration of MDAi (and/or higher treatment coverage and/or more frequent treatment; Stolk et al., 2015) or higher-than-appropriate thresholds of vector infectivity for deciding to stop interventions. An alternative explanation that has yet to be modeled, or considered explicitly in any guideline, is that continuing transmission/recrudescence may be a result of continuing parasite invasion from neighboring areas; i.e., there may be discordance between an intervention or evaluation area and the parasite transmission zone within which the area is located. In order to achieve sustainable elimination, transmission must be interrupted in the whole transmission zone, which needs to be effectively delineated to ensure that the whole zone is included in both interventions and evaluations.

The risk of parasite invasion is illustrated by two reports from river basins in Burkina Faso where transmission was considered to have been permanently interrupted. In the Comoé basin, retrospective analysis of recrudescence suggested that long-range vector movement was responsible, although immigration of infected people from outside the basin was not excluded. Ongoing transmission was confirmed *via* entomological assessments at 4/4 capture points (Koala et al., 2017; Koala et al., 2019). The second Burkina Faso report investigated microfilariae-positive individuals in the Upper Mouhoun, Nakambé, and Nazinon river basins and found all but 1 of the 31 infected individuals recently resided in neighboring Côte d’Ivoire (Nikièma et al., 2018). The remaining person had been previously identified as infected in 2008 (Nikièma et al., 2018). Thus, migration between an area with and an area without ongoing transmission accounted for 30/31 of the identified infected people.

The two cases described above point to immigrant parasites as the potential source of renewed transmission and emphasize the importance of knowing whether or not new infections are due to parasites which “immigrated” *via* vectors or humans for determining areas to be included in appropriate treatment strategies. Similarly, the estimated risk of immigration *via* humans or vectors is important for elimination programs to make cost–risk based decisions: i.e., evaluate the cost of stopping MDAi based on the risk of resurgence due to immigration from areas that do not yet meet stopping criteria versus the cost of continuing MDAi until these other areas also meet stopping criteria. Finally, such information can provide lessons for evaluations required for decisions to stop interventions, including delineation of evaluation areas, sampling criteria, or thresholds for MDAi cessation, which are all currently determined independent of vector abundance.

#### Lymphatic Filariasis

In 2012, 73 countries were regarded as LF endemic (Ramaiah and Ottesen, 2014), with 55 implementing MDA. By October 2019, 15 countries had been validated as having eliminated LF as a public health problem: Vanuatu, Tonga, Niue, Cook Islands, Republic of the Marshall Islands, Palau, Wallis & Futuna, Kiribati, Cambodia, and Vietnam in the WHO Western Pacific Region (WPRO); Sri Lanka, Maldives, and Thailand in the WHO South East Asian Region (SEARO); Egypt in the WHO Eastern Mediterranean Region (EMRO); and Togo in the WHO African Region (AFRO) (World Health Organization, 2019) (**Figure 2**).

While 15 countries have achieved validation of elimination so far (and some are nearing this milestone), there are countries where progress toward elimination has stagnated or reversed, including several in the Pacific where LF is transmitted by efficient day-biting *Aedes* vectors and where there may also be supplementary night-biting vectors (Ichimori and Graves, 2017). These countries include American Samoa, Samoa, French Polynesia, and Fiji.

LF is relatively heterogeneous in its distribution within countries, and thus the role of movement between intervention/evaluation units in maintaining or re-establishing transmission is a concern for many national LF elimination programs. Transmission zones in *W. bancrofti* are highly dependent on variation in vector density, survival, and competence for transmission and whether vector mosquitoes are diurnal, nocturnal, or sub-nocturnal. In areas with post-intervention recrudescence, there are sometimes infection “hot spots”: a localized area where prevalence is significantly higher than surrounding areas (e.g., American Samoa: Lau et al., 2014; Lau et al., 2016; Lau et al., 2017; Sri Lanka: Rao et al., 2014; Rao et al., 2017; Rao et al., 2018). Potential explanations for recrudescence include expansion of infections from hot spots when mosquitoes take up and transmit parasites to geographically nearby communities or when infectious people visit other communities, slow but insidious increase in frequency and intensity of transmission from widespread infections at low prevalence that were not detected or were considered (incorrectly) to be under the threshold for stopping MDA, or migrants from other endemic regions continue to introduce new parasites, if the evaluation unit is inappropriately small.

The role of population movement between countries and regions within countries in maintaining or re-establishing transmission is a concern for many national elimination programs. Continued surveillance post-MDA has been recommended by researchers when communities are in close proximity to cross-border regions with ongoing transmission (Ramaiah, 2013; Dorkenoo et al., 2018). The possibility that highly mobile migrant workers from Myanmar introduced and maintained LF transmission in Thailand, after effective MDA throughout most of the latter country, resulted in additional rounds of MDA in border communities and sparked epidemiological research on the connectivity between communities (Bhumiratana et al., 2010; Satimai et al., 2011; Bhumiratana et al., 2013; Toothong et al., 2015; Dickson et al., 2017). The influx of thousands of refugees from Haiti, where LF is hyperendemic, to Brazil, where LF transmission has been reduced and even eliminated in some states, has prompted screening and treatment of recent immigrants to reduce risk of re-introduction (Nunes et al., 2016; da Silva et al., 2017; Zuchi et al., 2017). Even in the remote islands of Samoa and American Samoa, models have shown that movement of people between countries could have a significant impact on the transmission of infectious diseases, including LF (Xu et al., 2018b). The role of movement of infected persons in the establishment and persistence of hot spots, and thus the appropriate choice of the size for implementation and evaluation units (both in terms of geographic size and total population) for LF programmatic activities needs to be critically evaluated for their impact on elimination progress. In contrast, long distance vector dispersal is not typically considered a major risk for increasing the distribution or prevalence of the LF parasite (Hapairai et al., 2013; Verdonschot and Besse-Lototskaya, 2014, but see Huestis et al., 2019).

Crucial questions to be addressed for sustainable LF elimination are thus: (1) what drives development of hot spots, and their persistence after MDA, (2) what are effective methods for post-MDA surveillance, when very low infection prevalence becomes difficult to detect, (3) how to determine appropriate target thresholds for successful interruption of transmission in different settings (e.g., areas with different mosquito vectors), (4) what strategies should be used to delineate intervention/evaluation units, (5) what role does travel/migration (i.e., population movement and connectedness) play in the transmission of parasites between countries and between regions within countries, and (6) how do transmission dynamics among regions affect planning for future MDA? To stop interventions and enter the “post-MDA surveillance” phase in a specific geographic area requires reasonable certainty that the probability of importation of parasites is too low to re-initiate transmission, i.e., that stopping criteria are met across the whole transmission zone. In other words, stopping MDA for LF also requires delineation of transmission zone boundaries.

## Parasite Population Genetics and Transmission Zones

Filarial nematodes have nuclear genomes on the order of ~90–100 Mbp, mitochondrial genomes of ~13–14 Kbp, and most, but not all, species obligately carry a *Wolbachia* endosymbiont that has its own genome of ~1 Mbp (Keddie et al., 1998; Keddie et al., 1999; Unnasch and Williams, 2000; Foster et al., 2005; McNulty et al., 2012; Ramesh et al., 2012; Desjardin et al., 2013; Cotton et al., 2016; Small et al., 2016; Chung et al., 2017). Sequencing, assembling, and annotating all three genomes for many helminth species, including the filariae, is the objective of continuing efforts, and the availability and assembly quality of parasite genomes will likely continue to increase rapidly. Currently, the *O. volvulus* genome has the highest quality assembly, with complete assemblies of mitochondrial and *Wolbachia* genomes and nuclear genome sequence scaffolds that are approaching whole chromosomes. Genome resources for this and other parasites can be found at https://parasite.wormbase.org/index.html.

With a few notable exceptions (Choi et al., 2016; Small et al., 2016; Doyle et al., 2017; Small et al., 2019), most genomic research for filarial nematodes has focused on predictions of gene function. The aim of many of these “functional” predictions is to identify targets for new drug discovery, usually in the absence of methods for functional genomic validation of targets. Given the time for moving from identification of potential drug targets to regulatory registration and WHO guidelines for new drugs, it is unclear to what extent drugs emerging from such research are going to be available for use by LF and onchocerciasis elimination programs.

In contrast, relatively little attention has been paid to the potential for the explosion of genomic information to support elimination efforts with *currently used* drugs or new applications of drugs such as moxidectin, which achieved regulatory registration with the U.S. Food and Drug Administration in 2018 and is undergoing additional studies for inclusion in WHO guidelines (Opoku et al., 2018). Understanding the geographic distribution of variation in susceptibility to those drugs—whether, for example, drug-naive populations already contain alleles for reduced susceptibility—may allow us to act to preserve susceptibility to those drugs. Population genomics analyzes whole-genome variation within and between parasite populations, identifying where that variation might impact drug efficacy (the evolution of drug resistance) or elucidating patterns of disease transmission (genetic epidemiology). We review applications of population genomics to informing when and where to cease treatment, on the quantifiable risk of recrudescence through migration of infected humans/vectors from other areas (*O. volvulus Population Genetic Structure* and *W. bancrofti Population Genetic Structure*), and on the long-term sustainability of MDA if poor response to drugs is heritable (*Parasite Population Genomics for Understanding Geographic and Temporal Variability in Drug Response*).

### Conceptual Approach

**Figure 4:**
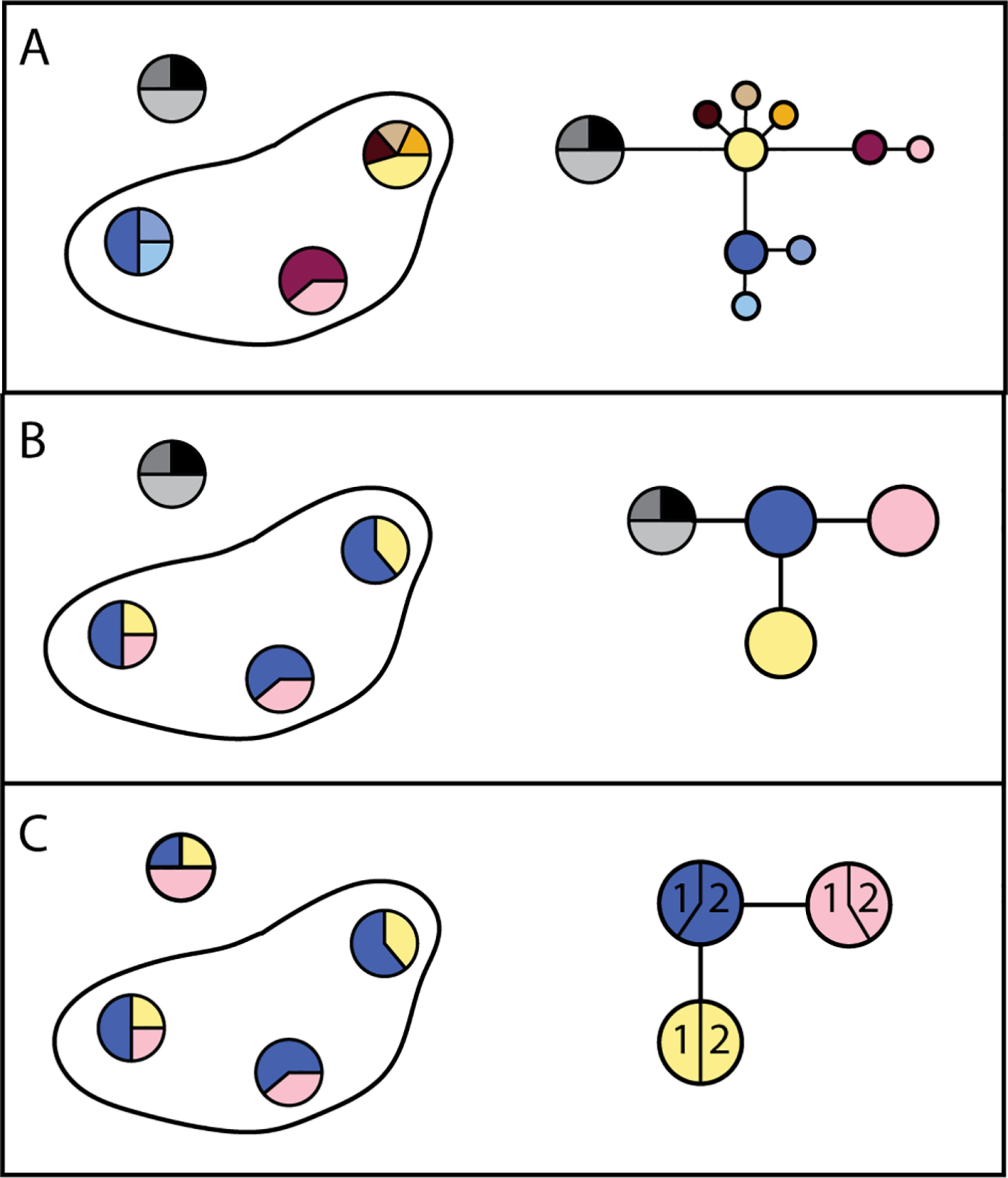
Alternate possibilities for distribution of genetic variation in parasites within a hypothetical country. **(A)** Haplotype frequencies (each unique sequence has a different color) from three locations within one country where nematodes were collected, and from a second country (haplotypes in grey), indicate all three locations are genetically distinct. The resulting haplotype network indicates sequence divergence between haplotypes. Taken together, these results would support the conclusion that there are three transmission zones in which parasites persist. **(B)** Haplotype frequencies in three locations where nematodes were collected indicate that they are genetically similar. The resulting haplotype network supports the conclusion that there is one transmission zone, and that recrudescence is local. **(C)** Haplotype frequencies in three locations indicate that they are genetically similar. The haplotype network indicates that recrudescence may have come from a migrant source, because haplotypes are shared between countries (1 and 2).

Genetic structure is the result of movement of parasites within, but not between, transmission zones: parasites within a transmission zone interbreed and are genetically more similar to each other than to parasites in another transmission zone, with whom they do not interbreed. The likelihood that parasites are from the same transmission zone, and thus likelihood of parasite transmission between two locations (invasion), can therefore be inferred from the degree of genetic relatedness between them (e.g., Blouin et al., 1995; Nadler, 1995; Criscione and Blouin, 2004; Small et al., 2014). Statistically significant genetic differentiation at neutral loci (i.e., loci which do not affect a phenotype subject to selection pressure) between two parasite samples indicates that they originate from two separate transmission zones (**Figure 4**). In contrast, high genetic similarity among parasites indicates either historical or ongoing gene flow (i.e., interbreeding), and that these more closely related parasites are from a single transmission zone, regardless of geographical scale. This genetic population structure—or lack of it—is the result of long-term patterns of transmission. Measures of population genetic structure could thus help programs assess the risk of resurgence associated with alternative scenarios on a longer time scale than intrinsically shorter-term decisions about where and whom to treat. Population genetic measures have proven to be informative for malaria epidemiology and control, particularly for investigating sources of transmission or the origins and prevalence of drug resistant strains (Jennison et al., 2015; Lo et al., 2017; Aydemir et al., 2018; Fola et al., 2018; Waltmann et al., 2018).

In population genetic analyses, sequence data are collected from parasites from multiple geographic locations. One approach to analyzing these data is to compare and cluster sequences based on the distribution of genetic variation across a geographic area: members of the same parasite population share similar patterns of genetic sequence variation because they have been interbreeding (e.g., Pritchard et al., 2000; Jombart et al., 2010). An alternative approach is to explore the history of parasite movement by simulating the expected distribution of genetic variation under different parasite migration histories, and comparing test statistics derived from these simulated data to test statistics calculated using the observed genetic variation (e.g., Laval and Excoffier, 2004; Peng and Kimmel, 2005). Development of analytical methods suitable for quantitative assessment of transmission of parasites within and among geographic regions is an active area of research, and more in-depth reviews of these methods can be found elsewhere (e.g., Schawbl et al., 2017; Wesolowski et al., 2018).

Prior to MDA, filarial parasite populations had a widespread, continuous distribution (e.g., onchocerciasis in Central/East Africa; **Figure 3**). Because MDA (or vector control) reduces or interrupts transmission, parasite population sizes fluctuate over time, and their geographic distribution becomes increasingly patchy. Parasite movement within a large geographic area in which parasite populations were reduced to zero only in some subareas can lead to transmission being re-established where it was previously interrupted. These re-established parasite populations and the parasite populations from which they derive form what in ecology and population genetics is called a “metapopulation” (Levins, 1969; Ariey et al., 2003; Churcher et al., 2008; Tadiri et al., 2018). Where there is a pattern of re-establishment post-intervention, parasites within a metapopulation are genetically similar, and the geographic range of this metapopulation defines the transmission zone.

Parasitic nematodes tend to have higher genetic diversity than vertebrates (e.g., Blouin et al., 1992; Grant and Whitington, 1994; Blouin et al., 1995; Hawdon et al., 2001; Prichard, 2001), possibly due to their complex life cycles, transmission epidemiology (Criscione et al., 2005), and large population size. Although the high genetic diversity in parasites may require a large sample size to ensure the data are representative, estimating population genetic structure does not require a complete, assembled genome. Researchers target genomic regions identified as sufficiently informative to reveal relatedness among individuals. For example, estimating genetic diversity and population structure using mitochondrial sequence data is well established in population genetics. The advantages of mitochondrial markers include that they are maternally inherited, have a higher mutation rate than the nuclear genome (10- to 17-fold; although this may be lower in species associated with *Wolbachia*; Cariou et al., 2017), have a high copy number relative to the nuclear genome in each cell (useful for DNA sequencing of samples with low concentration or degraded DNA), and do not generally recombine. Mitochondrial genomes upon which markers for population genetics can be built are also widely available across filarial nematodes, including *O. volvulus* (Keddie et al., 1998; Unnasch and Williams, 2000), *W. bancrofti* (Ramesh et al., 2012; Small et al., 2016), *B. malayi* (Williams et al., 2000), *L. loa* (Higazi et al., 2004), and the canine heartworm, *Dirofilaria immitis* (Hu et al., 2003).

There are advantages to conducting population genetic analyses that include nuclear markers: they are inherited from both parents, and the extent of recombination between markers can be informative about past demographic events such as reductions in population size or selection on heritable traits (both events cause markers to be linked: two or more DNA variants on a chromosome become associated more often than expected by chance). Nuclear variation has been estimated using partial sequences of multi-copy ribosomal genes or spacers, common repeat sequences, microsatellites, and fingerprinting using electrophoresis of randomly amplified DNA (RAPD). While these approaches have been useful due to their low cost compared to whole-genome sequencing, ease of experimental procedures, and straightforward comparisons between species (e.g., Blouin et al., 1995), capturing only a small part of genome variation underestimates diversity and can result in estimates of genetic variation with low information content (e.g., Keddie et al., 1999; Higazi et al., 2004). Analyses that utilize whole-genome sequencing data, rather than a few selected loci, can also identify the confounding effects of selection on estimates of population size, structure, and migration (see, e.g., Weigand and Leese, 2018). Increasing the information available per individual using whole-genome sequencing of either individuals or pools of individuals, and increasing the information available per population by increasing the total number of individuals sequenced, has improved the sensitivity and power of analyses, and will improve our understanding of disease transmission.

### *O. volvulus* Population Genetic Structure

*O. volvulus* population genetic structure is determined by the geographic range and movement of its hosts and vectors. The major blackfly vectors in Africa belong to a species complex (*S. damnosum s.l.*) divided into a number of subspecies based on cytotaxonomy (Boakye, 1993; Boakye and Meredith, 1993; Krueger and Hennings, 2006), although these subspecies are not all distinguishable using DNA sequence data (Tang et al., 1995; Tang et al., 1996). Variation in vector species distribution could influence *O. volvulus* population structure, because different species may influence transmission dynamics differently. Specific vector–parasite associations have been postulated (Duke, 1981; Basáñez et al., 2009). Blackflies from forest areas transmit more infective larvae of *O. volvulus* than flies prevalent in savannah areas (Cheke and Garms, 2013), and onchocerciasis-associated ocular damage is more common in savannah than in forest areas (Dadzie et al., 1990).

Because of this higher prevalence of onchocercal blindness in savannah compared to forest communities, early population genetics research focused on methods for differentiating “blinding savannah” and “non-blinding forest” parasite “strains.” Nuclear genome variation in the O-150 repeat family differentiated *O. volvulus* collected from forest and savannah communities in West Africa and Nigeria (Erttmann et al., 1987; Erttmann et al., 1990; Zimmerman et al., 1992; Zimmerman et al., 1994a; Ogunrinade et al., 1999), but not Uganda or Sudan (Fischer et al., 1996; Higazi et al., 2001). DNA sequences derived from a ribosomal internal transcribed spacer (ITS-2) could not distinguish forest and savannah *O. volvulus* from West Africa (Morales-Hojas et al., 2007). Analyses of nuclear genome sequences quantified admixture between parasites collected from different forest and savannah sites: parasites can have a mix of ecotype-associated markers (Choi et al., 2016), indicating that parasites from forest and savannah can and do interbreed and their respective transmission zones overlap.

**Figure 5:**
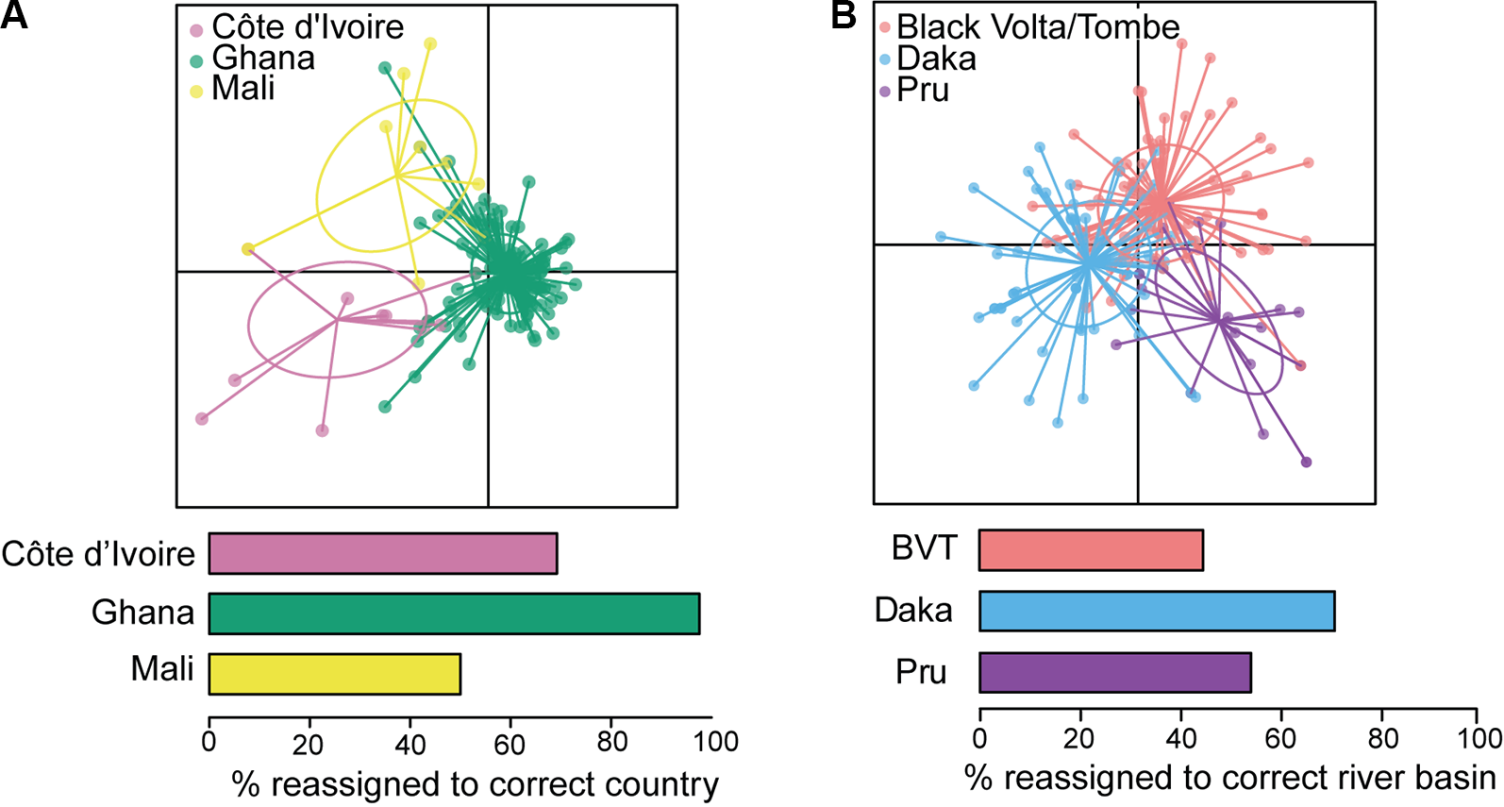
Analyses of mitochondrial sequence data from West Africa. **(A)** Discriminant analysis of principal components (DAPC; Jombart et al., 2010) indicates that Ghana is distinct from Côte d’Ivoire and Mali. **(B)** DAPC indicates that three river basins within Ghana should be considered a single transmission zone. BVT, Black Volta/Tombe. From Crawford et al. (2019).

Reconstruction of mitochondrial genomes of a small number of geographically diverse adult *O. volvulus* (Choi et al., 2016) has pointed to greater genetic diversity than previously reported (Zimmerman et al., 1994b; Keddie et al., 1999). Crawford et al. (2019) analyzed whole mitochondrial genomes of ~150 parasites from West Africa and demonstrated that *O. volvulus* populations are extremely genetically diverse and that the historical population size was likely very large. More importantly in the context of this review, a subset of mitochondrial markers could distinguish *O. volvulus* sampled in Ghana from those sampled in Mali or Côte d’Ivoire (via discriminant analysis of principal components; **Figure 5A**). Nuclear sequence data differentiated worms collected in Ghana from those collected in Cameroon, indicating that current, ongoing interbreeding between parasites from these two countries is unlikely. Therefore, they are from different transmission zones (Doyle et al., 2017). At an even larger geographic scale, admixture analysis of genetic sequence data demonstrated that American parasite populations are derived from African populations: competent vectors existed in areas where infected people from Africa were forcibly brought to work as slaves (Zimmerman et al., 1994b; Unnasch and Williams, 2000; Choi et al., 2016).

Analysis of population structure over shorter distances within a single ecotype has been conducted for parasites from different river basins. Nuclear genetic differentiation was clear between individual parasites from the Mbam and Nkam river basins in Cameroon, suggesting no (or limited) parasite transmission between them, and that, consequently, the Mbam and Nkam river basins belong to two different transmission zones (Doyle et al., 2017). In practical terms, this means that decisions to stop treatment in one basin may not need to take into consideration ongoing transmission in the other. In contrast, both nuclear (Doyle et al., 2017) and mitochondrial genomes showed little genetic differentiation among parasites from three river basins across an east-west transect of ~250 km in Ghana: the Daka river basin on the eastern side of Lake Volta, the Pru river basin on the western side, and the Black Volta/Tombe river basin to the north (**Figure 5B**; Crawford et al., 2019). These results are consistent with weak population structure between the three basins, and significant past or current gene flow between them. The epidemiological interpretation of this population structure is that transmission has occurred and likely continues to occur amongst these three river basins (Crawford et al., 2019). The Black Volta/Tombe, Pru, and Daka river basins should thus be considered a single transmission zone: cessation of treatment at any one location would require interruption of transmission throughout the zone to reduce risk of recrudescence.

The analyses of mitochondrial and nuclear variation presented here suggest that *O. volvulus* populations—and thus transmission zones—can cover large regions and different river basins. It remains to be seen to what extent these genetic estimates of transmission zones can be correlated with wind-facilitated vector movement, features of the local climate or landscape that impact fly prevalence because of breeding site availability, differences in transmission capacity of the local fly vector species, or factors impacting movement among regions by people such as low habitability, large distances, or seasonal migration of workers.

### *W. bancrofti* Population Genetic Structure

Within populations of *W. bancrofti*, genetic diversity (i.e., the number of polymorphic sites, regardless of genetic background) and haplotype diversity (i.e., the number of unique, contiguous sequences) appear to be comparable to *O. volvulus* (de Souza et al., 2014; Choi et al., 2016; Small et al., 2016; Doyle et al., 2017; Crawford et al., 2019; Small et al., 2019). Diversity may be higher where transmission rates are higher (Blouin et al., 1992; Blouin et al., 1995), because population sizes are expected to be larger where there is more active transmission, and larger population sizes result in greater retention of genetic diversity over time. Studies in Papua New Guinea (PNG; Small et al., 2013), Ghana (de Souza et al., 2014), and India (Thangadurai et al., 2006; Hoti et al., 2008b; Mahakalkar et al., 2017) have found high levels of genetic variation among and within *W. bancrofti* populations, and suggested higher similarities between geographically proximate villages. Consistent with expectations that higher transmission rates (and consequently, larger parasite population size) may increase genetic diversity, Hoti et al. (2008b) detected the highest genetic variability in densely populated, urban areas.

Genetic differentiation has been associated with geographic distance and other barriers to movement of people and vectors in Ghana (de Souza et al., 2014), India (Kumar et al., 2002; Thangadurai et al., 2006; Hoti et al., 2007; Patra et al., 2007), and Nepal (Adhikari et al., 2015). For example, genetic fingerprinting detected divergence among strains on either side of the Western Ghats mountain range in India, likely driven by the geographic barrier (Thangadurai et al., 2006). Genetic differentiation between parasites from different vectors was identified in the Andaman and Nicobar Islands (Dhamodharan et al., 2008), and a nocturnally sub-periodic strain from Thailand was genetically distinguishable from a nocturnally periodic Myanmar strain (Nuchprayoon et al., 2007). These genetic differences could be driven by geographic distance reducing gene flow or a consequence of divergent phenotypic adaptation to enhance transmission by different vectors. A significant challenge with these studies is that many of them used RAPD assays, yielding data which are difficult to compare between studies and have uncertain reproducibility (Penner et al., 1993; Davin-Regli et al., 1995; Pérez et al., 1998).

Genomic sequencing of *W. bancrofti* within populations has been limited to 20 individual worms from PNG (Small et al., 2016; Small et al., 2019) and 27 from three geographically widespread communities (Haiti, Mali, and Kenya; Small et al., 2019). Small et al. (2019) analyzed all 47 genomes and found genetic differentiation among all countries sampled, suggesting migration is low or non-existent at this spatial scale. Further analyses supported ancestral movement of parasites between Africa and Haiti, likely driven by the forced imprisonment and transport of people from Western and Central Africa to the Americas (Small et al., 2019). These efforts indicate sufficient genetic variation in *W. bancrofti* to detect gene flow. Advances in whole-genome sequencing of *W. bancrofti*, such as those made by Small et al. (2016; 2019), using either infective larvae dissected from laboratory-reared mosquitoes or using selective whole-genome amplification of single microfilaria isolated from blood samples, may increase the feasibility and accuracy of using molecular sequence data to identify and track transmission of LF. In particular, advances in laboratory methods that could enable sequencing from materials commonly collected during TASs (e.g., from rapid diagnostic tests: Rao et al., 2006; microfilariae slides: Bisht et al., 2006; and dried blood spots: Kumar et al., 2019), would in turn increase availability of sequence data in terms of the number of individual parasites per community and the number of communities, allowing accurate delineation of transmission zones and identification of immigrant parasites.

## Parasite Population Genomics for Understanding Geographic and Temporal Variability in Drug Response

### Conceptual Approach

One aim of population genomics is to identify genome variation associated with phenotypic variation in traits of interest. Because neither *O. volvulus* nor *W. bancrofti* can be cultured in the laboratory, this work requires correlations to be determined from worms collected from infected people and phenotyped for a trait (e.g., disease manifestation, variation in drug response) and then subsequently genotyped. For example, onchocerciasis disease severity and manifestations vary widely across Africa, with onchocerciasis-associated blindness more common in savannah regions (Remme et al., 1989; Dadzie et al., 1990), and severe onchodermatitis (Sowda, hyperreactive onchocerciasis) prevalent in Yemen and in one focus in Sudan (Mukhtar et al., 1998), but rare in other endemic areas (Al-Kubati et al., 2018). Similarly, the extent to which the level of skin microfilariae decreases after ivermectin treatment and the timing and extent of subsequent increases in skin microfilariae levels varies between individuals even in ivermectin-naive areas (Awadzi et al., 2014; Opoku et al., 2018).

**Figure 6:**
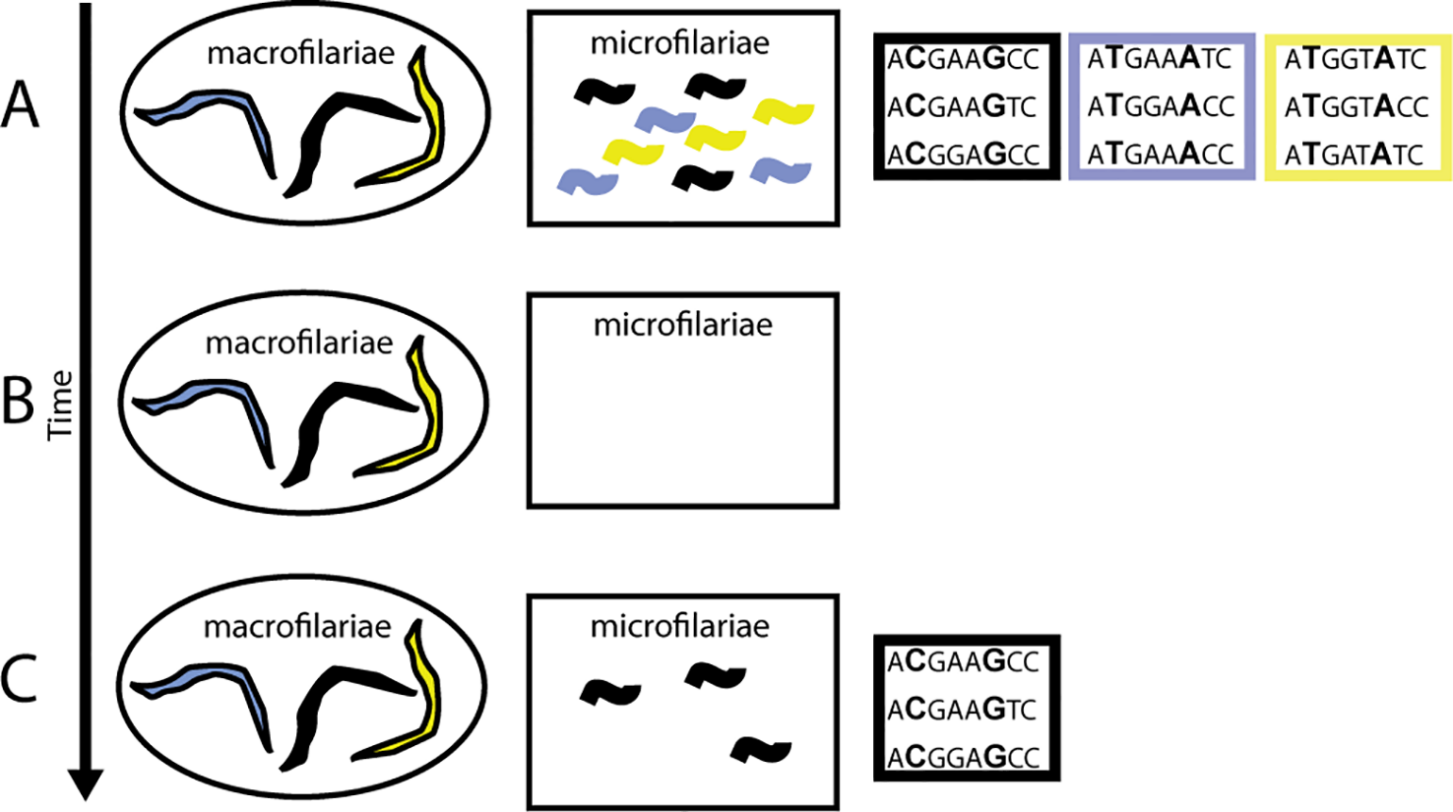
Impact of ivermectin on transmission when there is variation in worm response. **(A)** Pre-treatment phenotypic (colors) and genetic diversity within a person is high. **(B)** When a person is treated with ivermectin, microfilariae are cleared from the skin and the uteri of the worms. **(C)** Post-treatment, some worms begin reproducing earlier than others, giving them a transmission advantage (black phenotype). If this is a heritable trait, then a correlation between the phenotype and genotype arises; in this case, between both the C at position 2 and the G at position 6 in the sequence.

The challenge is determining whether there is a correlation between a well-identified phenotype (e.g., early resumption of female worms’ fertility after drug treatment) and a particular genotype (**Figure 6**). Identifying parasite population structure is a critical first step: it allows identification of genetic similarities and differences that arise because of degrees of relatedness between worms (interbreeding) and not because of genetic association with the parasites’ phenotype. Such genetic variants should be excluded from analyses of correlations between phenotypic and genotypic variation. Equally important, and perhaps more challenging, is that the phenotype needs to be well defined. For example, when comparing differences in pathology, manifestations might depend on whether the person was infected as a child or as an adult, on variation in the longevity of microfilariae or adult worms in different geographic regions (e.g., forest and savannah worms; see Duke, 1993), or on the genetic background of the human host driving specific immunologic reactions (e.g., Sowda; see Hoerauf et al., 2002).

### Defining Phenotypic Variation in Drug Response

The potential for the evolution of resistance to either macrocyclic lactones (such as ivermectin) or benzimidazoles (such as albendazole) in filarial nematodes infecting humans has been a concern from the start of MDA programs (e.g., WHO Expert Committee on Onchocerciasis Control 1993 and WHO, 1995), because of demonstrated evolution of resistance in parasites of domestic animals (Kaplan, 2004; Wolstenholme et al., 2004; Wolstenholme et al., 2015). However, identifying drug resistance in human-infecting filarial parasites that cannot be cultured in the laboratory is challenging, and quantifying genetic association with a phenotype is completely dependent on identifying the correct phenotype.

In *O. volvulus*, the phenomenon of “suboptimal response” to ivermectin (SOR) was identified during research into reasons for higher-than-expected prevalence of infection and skin microfilariae levels in areas under long-term MDAi in Ghana. SOR is defined as the resumption of microfilariae production by adult female worms following ivermectin treatment earlier than considered typical, resulting in detectable levels of skin microfilariae 80–90 days post-treatment and the presence of viable embryos and microfilariae in the uteri of female worms obtained through nodulectomy (Awadzi et al., 2004a; Awadzi et al., 2004b). Since reasons such as low ivermectin blood concentrations were excluded, Awadzi et al. attributed SOR to low susceptibility to the so-called “embryostatic” effect of ivermectin, resulting in developmental stages of microfilariae in the uteri 90 days after treatment (see also Grant, 1994). The progeny of these worms would be available for transmission earlier and for longer between rounds of MDAi than progeny of susceptible worms releasing microfilariae later (**Figure 6**). If low susceptibility to the “embryostatic” effect is heritable, the prevalence of SOR parasites may increase over time. While increasing prevalence of SOR worms is not expected to impact effectiveness of MDAi for control of onchocerciasis as a public health problem (because SOR does not change post-treatment disappearance of microfilariae from the skin—the “microfilaricidal effect”), it would negatively impact elimination, which depends on progressively lower skin microfilariae available for transmission. Since the initial description of SOR, further investigations of SOR in Ghanaian and Cameroonian areas with different ivermectin treatment histories (e.g., Osei-Atweneboana et al., 2007; Osei-Atweneboana et al., 2011; Pion et al., 2013; Nana-Djeunga et al., 2014) have shown that SOR parasites are detectable even where MDAi occurs biannually (Frempong et al., 2016). Modeling showed large inter-individual variability in skin microfilariae repopulation rates among individuals from Ghana, Liberia, Sierra Leone, and Guatemala treated one to five times with ivermectin (Churcher et al., 2009). None of the individuals from Ghanaian communities who received 10–19 rounds of ivermectin treatment (Osei-Atweneboana et al., 2007) had a higher repopulation rate higher than the highest observed in the control data set (skin microfilariae repopulation rate after the first ivermectin treatment among individuals from Ghana, Liberia, Sierra Leone, and Guatemala), but analysis by village showed significantly higher repopulation rate for some villages and for one previously untreated village in the same area (Churcher et al., 2009) This supports the conclusion that SOR parasites are present in ivermectin-naive communities and may increase *via* selection by ivermectin treatment. Furthermore, variability of response to ivermectin measured by skin microfilariae levels over time after a single dose of ivermectin was found among individuals from ivermectin-naive areas in Ghana, Liberia, and the Democratic Republic of Congo: the skin microfilariae kinetics in some of these individuals met the SOR criteria used in other studies (Awadzi et al., 2004a; Awadzi et al., 2004b; Opoku et al., 2018). It is unknown whether these ivermectin-naive areas belong to the same transmission zones as other nearby areas with ongoing MDAi. Together, these data support the conclusion that *O. volvulus* populations consist of worms with variable susceptibility to the embryostatic effect of ivermectin. Based on these data, the phenotype for genomic analysis is the reproductive status of individual female worms collected from people 80–90 days post-ivermectin treatment: female worms that respond well to ivermectin should not have post-fertilization embryonic stages, while those considered SOR have early resumption of fecundity, and thus already have microfilariae in uteri (Osei-Atweneboana et al., 2011; Nana-Djeunga et al., 2014; Doyle et al., 2017).

In *W. bancrofti*, reasons underlying continued persistence of transmission are challenging to determine. So far persistence has not yet been associated with heritable variability in parasite drug response. Density-dependent effects on vector response to infection and vector competence play a role in continuous, low-level transmission, depending on the vector species: iL3 development is proportionally higher at lower microfilariae densities in some vector species, while in other vectors, fewer iL3 develop when many microfilariae are ingested (Pichon, 2002; reviewed in Graves et al., 2016). These differences change transmission dynamics during MDA in ways highly dependent on specific vector species that may favor greater vector efficiency when microfilaremia is low.

### Defining Correlations Between Phenotypic and Genotypic Variation for Drug Response

Several point mutations in the target of albendazole, β-tubulin, lead to drug resistance in nematodes (Kwa et al., 1994; Grant and Mascord, 1996; Silvestre and Cabaret, 2002; Silvestre and Humbert, 2002), and at least one of these has been identified in *W. bancrofti* (Schwab et al., 2005). Increases in alleles associated with benzimidazole resistance have been detected after MDA (Schwab et al., 2005), and modeling suggests that further MDA treatments would lead to selection *via* an increase in the frequency of these alleles (Schwab et al., 2006; Schwab et al., 2007). However, analysis of natural variation in response to benzimidazole anthelmintics in the free-living nematode *Caenorhabditis elegans* has shown that many other loci contribute to and modify variation at the β-tubulin locus in this species (Hahnel et al., 2018), and it is clear from analysis on benzimidazole resistance in veterinary helminths that β-tubulin–mediated resistance is also subject to modification by as yet unidentified loci (Doyle and Cotton, 2019).

With respect to DEC, this drug is not 100% effective against LF (Eberhard et al., 1988; Eberhard et al., 1991), suggesting that variation in DEC susceptibility exists and could lead to the development and spread of drug resistance if poor susceptibility is both heritable and mating and recombination allow for assortment among alleles (Grant, 1994; McCarthy, 2005; Cobo, 2016).

The view that has emerged from single-candidate gene association studies in helminths is that response to macrocyclic lactones such as ivermectin (a glutamate-gated chloride channel agonist) is a complex and highly variable phenotype, affected by multiple genes across the genome (e.g., Dent et al., 2000; Ardelli et al., 2009; Yan et al., 2012; Ardelli and Prichard, 2013; Menez et al., 2016), with poor repeatability between studies, strains, and species. A single-candidate gene approach has been applied to ivermectin SOR in *O. volvulus* by sequencing genes that might contribute to ivermectin resistance in other nematodes, and comparing differences between communities that have never received ivermectin and those that have, or between microfilarial pools collected before and after treatment (Ardelli and Prichard, 2004; Ardelli et al., 2005; Eng and Prichard, 2005; Eng et al., 2006; Ardelli and Prichard, 2007; Osei-Atweneboana et al., 2012). If a gene contributes to ivermectin response, the expectation is that alleles that decrease sensitivity to ivermectin would be more common in worms from areas that have had many rounds of MDAi than in worms from ivermectin-naive areas, or that they would be more common in microfilariae collected shortly after ivermectin treatment than before treatment, or would be more common in adult females that have resumed reproduction within 80–90 days of treatment (the SOR phenotype). Genes identified using this approach include β-tubulin (Eng and Prichard, 2005; Bourguinat et al., 2006; Eng et al., 2006; Bourguinat et al., 2007; Osei-Atweneboana et al., 2012) and members of the ATP-binding cassette transporter family (Ardelli and Prichard, 2004; Ardelli et al., 2005; Eng and Prichard, 2005; Ardelli et al., 2006a; Ardelli et al., 2006b; Ardelli and Prichard, 2007).

However, this approach faces one of the biggest challenges in detecting selection: the frequency of an allele can increase or decrease due to random chance rather than selection. In particular, if a population decreases in size, as might be expected as transmission decreases during successive rounds of MDA, the chance that changes in allele frequencies are stochastic rather than deterministic increases: the genetic diversity overall decreases (e.g., *Schistosoma mansoni*: Coeli et al., 2013), but the fate of individual alleles is unpredictable, and some may increase due to genetic drift caused by population decline. Another challenge is that, prior to treatment, parasite populations can be quite large (i.e., they have a high effective population size; Blouin et al., 1995; Blouin et al., 1999; Zhou et al., 2013; Rodelsperger et al., 2014; Jan et al., 2016), which means they have ample genetic variation, so genetic variation contributing to a poor response phenotype may be present at different frequencies and at different loci in different parasite populations (e.g., *Haemonchus contortus*, Gilleard and Redman, 2016). Furthermore, once MDA begins, mating and recombination could create new combinations of alleles from different genes, which may also decrease response to ivermectin. In other words, good responders may be carriers of alleles that, in the right genomic background, could contribute to poor response in drug naive populations, and vice versa. Selection then acts on “standing” genetic variation that was present in the population before the selection pressure began. This mechanism for selection has been called a “soft sweep” (e.g., Hermisson and Pennings, 2005; Przeworski et al., 2005; Pritchard and Di Rienzo, 2010; Ferrer-Admetlla et al., 2014; see also Doyle et al., 2017) and is notoriously difficult to detect because (1) each variant alone could have a weak effect on phenotype, and thus difficult to distinguish statistically from background noise (random variation), and (2) many techniques for detecting selection rely on correlated, linked variants being physically near to the causative mutation, but in a soft sweep, this linkage is often weak (Pennings and Hermisson, 2006). The technical and conceptual weaknesses of candidate gene approaches specifically in the context of anthelmintic response was reviewed recently (Doyle and Cotton, 2019).

Genome-wide approaches are, therefore, required. Doyle et al. (2017) examined female *O. volvulus* macrofilariae from Ghana and Cameroon that were “good responders” and “suboptimal responders” to the embryostatic effect of ivermectin, determined by the absence or presence of stretched microfilariae in the uteri. Females were pooled by response phenotype, sequenced, and genetic variants correlated with phenotype identified. Genetic variants clustered in 31 quantitative trait loci (QTLs) with genes for molecular pathways involved in neurotransmission, development, and stress response. None of these QTLs contained genes previously proposed by single candidate association studies. In addition, some loci associated with SOR to ivermectin in worms from Ghana were different from loci associated with SOR in worms from Cameroon. They concluded that ivermectin response is a polygenic, quantitative trait, subject to soft sweeps of pre-existing QTLs, rather “hard selection” on rare, resistance-conferring mutations. Since populations possess different standing variation, selection may act on different loci in different populations (Doyle et al., 2017). Genome-wide studies of veterinary nematodes generally support the “soft sweep QTL” model for response to macrocyclic lactones (Bourguinat et al., 2015; Redman et al., 2015; Choi et al., 2017; Doyle et al., 2019), and likewise have not identified any of the candidate genes described above.

This “soft sweep QTL” model that has emerged from genome-wide association studies of ivermectin response leads to the following predictions for control and elimination of human filariases:

1. Substantial natural variation in ivermectin (and perhaps benzimidazole or DEC) response may exist within and between populations at the beginning of MDA. Therefore, in some populations, MDA could reduce transmission more efficiently than in others, ultimately leading to elimination success in some populations but not others. Genetic markers that predict drug response can first be defined (as has been done for *D. immitis* in the USA; Bourguinat et al., 2015; Bourguinat et al., 2017; Ballesteros et al., 2018) and then used to identify “at-risk” populations in which the initial SOR allele frequency is high enough to prevent elimination by MDA alone.
2. Selection for increased SOR allele frequency will likely occur *via* soft sweeps, making detection of underlying genetic changes more challenging. That said, a small panel of SNP alleles can predict treatment response in *D. immitis* (with the caveat that the population structure of *D. immitis* in these studies is not reported; Bourguinat et al., 2015; Bourguinat et al., 2017; Ballesteros et al., 2018).
3. Ivermectin response appears to be a highly complex phenotype, and changes in the molecular targets of ivermectin may not be the cause of SOR. For example, selection could favor worms with a faster rate of embryonic development, or resumption of microfilariae production might be driven not by female recovery from an embryostatic effect, but by recovery of males and thus earlier insemination, or by recovery of one or both sexes from an effect that impairs mating. The possibility that the embryostatic effect of ivermectin is a male- rather than female-mediated phenomenon has not been tested in *O. volvulus* or LF, but suppression of embryogenesis in *D. immitis* appears to be due to effects of macrocyclic lactones on the reproductive competency of male and not female worms (Lok et al., 1995). This has implications for how the drug response phenotype should be defined.
4. It is now clear that variation in drug response is heritable. If SOR alleles are initially present and selected for, even in only some communities undergoing MDAi, SOR could spread throughout the whole transmission zone (including to hypoendemic areas not initially included in MDAi). An important secondary advantage to identifying transmission zones, then, is that the potential risks and time frame of the spread of a SOR genotype to connected communities could be quantified.

## Development of Population Genomics–Based Tools to Inform Elimination Program Decisions

A wide range of DNA-based molecular tools have been developed for detecting the presence of parasites in vectors and the human host using skin, blood, plasma, and even urine (e.g., Zimmerman et al., 1993; Rao et al., 2006; Rodriguez-Pérez et al., 2006; Hoti et al., 2008a; Gass et al., 2012; Alhassan et al., 2015; Owusu et al., 2015; Lau et al., 2016). The addition of information on transmission gained from genomic analyses of parasites/vectors to data on prevalence and intensity could significantly improve the basis for elimination program decisions on whether to stop or continue interventions, determine causes for recrudescence post-intervention and thus appropriate subsequent interventions, and determine optimal post-intervention surveillance frequencies commensurate with the risk of resurgence based on a cost–risk evaluation.

Methods to obtain and analyze genome sequences require training and equipment not available to many laboratories in endemic countries. However, once the required basic genome research has been done, fast and relatively inexpensive methods can be developed suitable for use in at least one laboratory in an endemic country, possibly supported by one or more “reference laboratories.”

### Tools for Defining Transmission Zones and Identification of Migrants

The “genetic signatures” of an interbreeding parasite population constitute markers that allow delineation of transmission zones as “the natural ecological and epidemiological unit for interventions” for onchocerciasis or the implementation and evaluation units for LF. In conjunction with transmission models that allow modeling the impact of immigrant parasites on long-term infection prevalence, the size of these zones can inform cost–risk analysis for stop-treatment decisions as well as when and where to conduct entomological evaluations or TAS, dependent on the risk of resurgence that programs are willing to accept.

The advantage of DNA-based transmission zone markers is that they can be designed to be highly specific. The first step is identifying variant sites informative for population structure; i.e., are polymorphic and more common or fixed (and thus have different frequency-based likelihoods) in genomes of parasites from one area compared to another. For example, Crawford et al. (2019) identified mitochondrial polymorphic sites that could differentiate *O. volvulus* collected in Ghana from countries farther west with high statistical likelihood. Once such variant sites have been identified for specific geographic areas, parasite genomes from other areas need to be analyzed to determine whether the same variants are informative, or whether additional sites are necessary to infer the extent of interbreeding. Once analysis is completed for parasites from across their distribution, a minimum set of polymorphic sites can be defined to predict the transmission zone of origin for any given worm.

This process may sound daunting, but methodology developed over the past years is efficient and amenable to further optimization. Skin snips or infected/infective *Simulium* or *O. volvulus* adults from many areas in Africa are available in the ESPEN laboratory in Ouagadougou, national laboratories, and research centers. LF parasites, primarily on slides of blood films and in dried blood spots, are available from endemic areas in national laboratories and research centers.

Developing inexpensive methodologies for routine use in each endemic country is the next challenge. High resolution melt (HRM) analysis has been proposed as a species identification tool for *Onchocerca* spp. collected from blackflies (Doyle et al., 2016) and could potentially be extended as a genotyping tool. The advantage to using vectors is that, in the case of *Simulium* spp., vector collection is already an integral part of the process for stop-treatment decisions, invasive sampling of people would not be required, and (if sampling strategies are well designed) vectors would include a representative sample of parasites from many different human hosts.

### Tools for Monitoring Drug Susceptibility

Ideally, control/elimination programs would have reference data on pre-MDA/early-MDA variability of parasite drug response and an easy-to-use, cost-effective, and non-invasive tool for monitoring changes in response phenotype frequency. Detecting changes while the frequency of poor responder alleles is low would allow alternative control/elimination strategies to be put in place (Grant, 2000) and enable evidence-based decisions on monitoring strategies. Some moves toward these ends have been initiated (e.g., Ardelli and Prichard, 2004; Eng and Prichard, 2005; Bourguinat et al., 2006; Bourguinat et al., 2008; Hoti et al., 2009) but have yet to result in practical application. Research for methods development for monitoring potential emergence of resistance to ivermectin in *O. volvulus* were initiated by the UNDP/World Bank/WHO Special Programme for Research and Training in Tropical Diseases (TDR) and OCP in the early 1990s (WHO Expert Committee of Onchocerciasis Control (1993) and WHO, 1995; UNDP/World Bank/WHO Special Programme for Research & Training in Tropical Diseases, 1997). TDR also initiated characterization of parasite drug response variability before or early during MDA (Walker et al., 2016; Halder et al., 2017), research which continues with Wellcome Trust funding leveraged with this initial TDR investment. Alternative strategies for onchocerciasis elimination include increased MDAi frequency, complementary vector control, test-and-treat strategies, and new drugs (African Programme for Onchocerciasis Control, 2015; Boussinesq et al., 2018; Verver et al., 2018; Horstick and Runge-Ranzinger, 2018).

Currently, screening communities for *O. volvulus* with low ivermectin susceptibility requires either collection of skin snips before and after ivermectin treatment to determine skin microfilariae levels, or post-treatment nodulectomies to determine the reproductive status of individual female worms. Genetic analysis of microfilariae collected *via* skin snips at a single post-treatment time point, or larvae in vectors, could significantly improve monitoring capacity. While the molecular mechanisms that contribute to variation in drug response are of significant interest, identifying anonymous genetic variants associated with drug response phenotype is sufficient for development of tools for monitoring, even while research into the mechanisms is ongoing.

Genetic variation associated with ivermectin response in *O. volvulus* has been identified in parasites from Ghana and Cameroon (Doyle et al., 2017). These results have not yet been validated with single-worm genomic analysis, and whether the response-associated genetic variants identified are shared across populations from other areas in Africa is untested. Compared to development of transmission zone markers, development of ivermectin response markers is much more challenging, since large numbers of parasites with known response phenotype from different geographic areas are needed to identify, validate, and test the predictive value of genetic variants associated with response phenotype.

Identification of a panel of relatively few variants (e.g., < 50) would pave the way for developing a simple PCR-based or loop-mediated isothermal amplification (LAMP) surveillance tool for use with parasites in vectors or skin snips by endemic country laboratories. This core set of variants would define the QTLs that are both necessary and sufficient for SOR to develop in *O. volvulus* and may, therefore, shed light on the as yet unexplained mechanism by which ivermectin exerts its embryostatic effect.

MDA for *W. bancrofti* uses albendazole, alone or in combination with DEC and/or ivermectin. A PCR-based assay for one single nucleotide polymorphism that leads to resistance to benzimidazoles has been developed, so that monitoring for allele changes in treated communities can occur (Hoti et al., 2009). However, while variation in response to drug therapies for LF has been reported (e.g., Eberhard et al., 1988; Eberhard et al., 1991), to date a clear definition of an LF SOR has not been reported (Irvine et al., 2017). A clear, and preferably quantitative, definition of poor response is an essential prerequisite for development of response genetic markers in LF.

### Models Incorporating Population Genetics to Estimate Risk of Recrudescence From Migration or Poor Drug Response

There is a long history of the use of onchocerciasis and LF transmission models such as EPIONCHO and ONCHOSIM (reviewed in Stolk et al., 2015; Basáñez et al., 2016) and EPIFIL, LYMFASIM, and TRANSFIL (reviewed in Smith et al., 2017) to inform control program decisions. These models differ in whether they are deterministic or stochastic and whether they are population- or individual-based, and can be parameterized to match the characteristics of a particular community, including endemicity, variation in biting rates, or treatment coverage, such that effects of different treatment strategies on transmission can be explored. However, these programs currently cannot model movement of parasites from one geographic area to another (via people or vectors), spatial heterogeneity of endemicity or elimination program implementation, or transmission of parasites with variable drug susceptibility. Thus, although these models have been useful in estimating declines of parasite infection prevalence and intensity over a control campaign, they do not realistically model variation in parasite drug susceptibility, human/vector movement among areas, or long-term population processes that could impede reaching elimination thresholds or contribute to medium- to long-term recrudescence after MDA discontinuation.

To address these limitations, TDR funded extension of transmission models for preventive chemotherapy-controlled diseases. Coffeng et al. (2017) incorporated evolution of changes in drug susceptibility in *O. volvulus* (and other parasites) into a stochastic, individual-based model, and McCulloch et al. (2017) adapted an infection-intensity model, EPIONCHO, into a patch framework for onchocerciasis, incorporating differences in endemicity and treatment between two adjacent geographic areas, or “patches,” and people and vector migration between patches. When simulating migration of seasonal workers from a patch where transmission is ongoing to a patch where MDAi had interrupted transmission, modest levels of migration (10% of the adult population move for between 1 and 12 months) were sufficient to re-establish transmission with a lag time of 5–10 years, and pre-MDA prevalence was reached within 30–50 years in the absence of renewed MDA.

Data from Burkina Faso showed a significant delay between cessation of interventions and recrudescence (Koala et al., 2017; Koala et al., 2019), suggesting that the predicted time frames for recrudescence modeled by McCulloch et al. (2017) are realistic. Such delays are not unexpected considering that parasite invasion may initiate transmission at a low level, and transmission takes time to become detectable. A model incorporating population genetic structure data, allowing simulation of vector and human movement–mediated parasite transmission between different geographic areas, could provide estimates of the risk and time frame of resurgence in one area where treatment cessation is planned while transmission is above stop-treatment thresholds elsewhere. National programs could obtain a measure of risk for stopping interventions given migration and an estimated time frame for resurgence, decide whether to intensify interventions in areas that are potential source areas of invasion, and plan cost-effective, post-MDA surveillance strategies.

A model parameterized for a specific region can be informative for elimination programs by exploring how variation in treatment coverage, changes in human demographic and travel patterns, vector control and migration patterns, or other factors will affect progress toward and post-intervention sustainability of elimination within different areas in a region. Such a spatially explicit, agent-based modeling framework was developed to explore LF transmission in the Samoan Islands (GEOFIL; Xu et al., 2018a). This type of model incorporates detailed information about individuals within the community and their connectivity, and simulates changes in characteristics such as age, physical location, and infection status. GEOFIL predicts spatial variation in LF infection prevalence across American Samoa over space and time. The model can contribute toward understanding transmission dynamics in areas of high infection prevalence (hot spots) which can help determine effective surveillance strategies for identifying hot spots, and how best to manage them. Incorporation of information from genomic analyses could enhance the utility of such models for elimination decisions. An agent-based framework does require significant data on individual people, information not available for many (or most) communities, and it will be valuable to see how lessons from a well-characterized system can be applied more broadly to areas where LF has re-emerged post-MDA.

## Conclusions and Recommendations

Population genomic analyses of *O. volvulus* and *W. bancrofti* show high levels of genetic variation consistent with historically large population sizes (> 10^5^). Genetic variation is spatially structured; parasite populations are genetically distinct, indicating that they are not interbreeding. For *O. volvulus*, genomic data from West Africa indicate that the geographic areas across which parasites interbreed—transmission zones—can cover hundreds of kilometers and extend beyond the boundaries of a “river basin, or a major section of a river basin” (African Programme for Onchocerciasis Control/World Health Organization, 2010). This large-scale structure is reflected in the pattern of onchocerciasis endemicity in Africa, with a mosaic of hypo-, meso-, and hyperendemic areas (**Figure 3**). For *W. bancrofti*, transmission zones are larger than expected based on the focal nature of many mosquito vector populations, consistent with the increasingly recognized role that movement of infected people plays. In epidemiological terms, genetic data indicate that significant transmission must have occurred (and likely to still be occurring) over tens to hundreds of kilometers.

We propose that long-range transmission *via* infected people and/or infective vectors has epidemiological significance for elimination programs that it does not have for control programs. The latter plan to continue treatment indefinitely, and consequently, the effect of occasional or continual long-range transmission, is suppressed by continuing MDA. In contrast, elimination programs stop treatment once relevant criteria have been met within a defined area, assuming transmission has been interrupted permanently. Without MDA, occasional or low but continual parasite transmission from outside an intervention area may result in recrudescence. Population genetic analyses can predict whether the potential for gene flow is epidemiologically significant. Consequently, elimination programs need to define the boundaries of the entire extended geographic area over which parasite transmission occurs and continue treatment until transmission is interrupted over the entire area. From an operational perspective, population genomic statistics do not differentiate between long-range transmission due to movement of vectors and that due to movement of people, and are thus informative independent of the cause of parasite migration.

What research and development are required to allow elimination programs to take advantage of population genomics? One clear need is gathering more (and better quality) sequence data across a greater geographical range, based on improved methods which allow sequencing from parasite material already available or routinely collected (blackflies or skin snips for onchocerciasis, and mosquitoes or blood samples for LF). Testing and development of analytical methods for quantifying parasite migration based on genetic, entomological, and epidemiological data, and their optimal application to transmission zone delineation, is critical. Tools not requiring specialized laboratory equipment must be developed for use by national programs to include in MDA and stop-MDA evaluations. These tools would allow investigation of the source of infections/transmission identified during post-treatment surveillance, facilitate monitoring for changes in the frequency of parasites with SOR to MDA, and promote planning cost-effective solutions. Comparable population genomic data for vectors would be a useful adjunct to differentiate between vector and human movement as sources of long-range transmission to inform appropriate interventions. Current transmission models need to be enhanced to allow modeling of areas with different endemicity, treatment history, progress toward elimination, heritable drug resistance, and parasite gene flow, to better estimate the risk and time frame of recrudescence when treatment is to be stopped in one area while transmission is continuing in another. Finally, the research and development outputs can improve procedures and criteria for cessation of treatment, including estimating risks of recrudescence to inform cost–risk assessment-based stop-treatment decisions and appropriate post-treatment monitoring strategies.

## Author Contributions

SH, KC, AK, PG, and WG wrote the first draft. SH, AK, KC, PG, MB, CL, DB, and WG critically reviewed, edited, and approved the final version of the manuscript. The authors alone are responsible for the views expressed which do not necessarily represent the views, decisions, or policies of the institutions with which the authors are affiliated.

## Funding

TDR, the Unicef/UNDP/World Bank/WHO Special Programme for Research and Training in Tropical Diseases provided the funds for open access of this review and support for SMH (B80149 and B80153). KEC was supported by an Australian Government Research Training Program (RTP) Scholarship.

## Conflict of Interest

AK works for TDR, the Unicef/UNDP/World Bank/WHO Special Programme for Research and Training in Tropical Diseases.

The remaining authors declare that the research was conducted in the absence of any commercial or financial relationships that could be construed as a potential conflict of interest.

The handling editor declared a past co-authorship with one of the authors WG.
